# The association of COVID-19 occurrence and severity with the use of angiotensin converting enzyme inhibitors or angiotensin-II receptor blockers in patients with hypertension

**DOI:** 10.1371/journal.pone.0248652

**Published:** 2021-03-18

**Authors:** Mingfei Li, Ying Wang, Ndindam Ndiwane, Michelle B. Orner, Natalia Palacios, Brant Mittler, Dan Berlowitz, Lewis E. Kazis, Weiming Xia

**Affiliations:** 1 Center for Healthcare Organization and Implementation Research (CHOIR), Bedford VA Healthcare System, Bedford, Massachusetts, United States of America; 2 Department of Mathematical Sciences, Bentley University, Waltham, Massachusetts, United States of America; 3 Department of Public Health, Zuckerberg College of Health Sciences, University of Massachusetts Lowell, Lowell, Massachusetts, United States of America; 4 Geriatric Research Education Clinical Center, Bedford VA Healthcare System, Bedford, Massachusetts, United States of America; 5 Department of Medicine, The University of Texas Health Science Center at San Antonio, San Antonio, Texas, United States of America; 6 Department of Health Law, Policy and Management, Boston University School of Public Health, Boston, Massachusetts, United States of America; 7 Department of Pharmacology and Experimental Therapeutics, Boston University School of Medicine, Boston, Massachusetts, United States of America; National Yang-Ming University, TAIWAN

## Abstract

**Background:**

A number of studies have reported the association between the use of angiotensin-converting enzyme inhibitor (ACEI) and angiotensin-II receptor blocker (ARB) medications and the occurrence or severity of coronavirus disease 2019 (COVID-19). Published results are inconclusive, possibly due to differences in participant comorbidities and sociodemographic backgrounds. Since ACEI and ARB are frequently used anti-hypertension medications, we aim to determine whether the use of ACEI and ARB is associated with the occurrence and severity of COVID-19 in a large study of US Veterans with hypertension.

**Methods:**

Data were collected from the Department of Veterans Affairs (VA) National Corporate Data Warehouse (VA-COVID-19 Shared Data Resource) between February 28, 2020 and August 18, 2020. Using data from 228,722 Veterans with a history of hypertension who received COVID-19 testing at the VA, we investigated whether the use of ACEI or ARB over the two years prior to the index date was associated with increased odds of (1) a positive COVID-19 test, and (2) a severe outcome (hospitalization, mortality, and use of intensive care unit (ICU) and/or mechanical ventilation) among COVID-19-positive patients. We used logistic regression with and without propensity score weighting (PSW) to estimate the odds ratio (OR) and 95% confidence interval (95% CI) for the association between ACEI/ARB use and a positive COVID-19 test result. The association between medication use and COVID-19 outcome severity was examined using multinomial logistic regression comparing participants who were not hospitalized to participants who were hospitalized, were admitted to the ICU, used a mechanical ventilator, or died. All models were adjusted for relevant covariates, including demographics (age, sex, race, ethnicity), selected comorbidities, and the Charlson Comorbidity Index (CCI).

**Results:**

The use of ACEI significantly decreased the odds of a positive COVID-19 test among Veterans with hypertension (OR = 0.917, (0.887, 0.948) and OR = 0.926, (0.894, 0.958) with PSW). The use of ACEI, but not of ARB, was also associated with significantly increased odds of using mechanical ventilators (OR = 1.265, (1.010, 1.584) and OR = 1.210, (1.053, 1.39) with PSW) among all COVID-19 inpatients compared to outpatients.

**Conclusions:**

In this study of Veterans with hypertension, ACEI was significantly associated with decreased odds of testing positive for COVID-19. With the exception of the association of ACEI with a small non-clinically-important increase in the odds of using mechanical ventilators, neither ACEI nor ARB was found to be associated with clinical severity or mortality among COVID-19-positive Veterans. The results of this study need further corroboration and validation in other cohort samples outside the VA.

## Introduction

The coronavirus disease 2019 (COVID-19) pandemic has spread across the globe at an unprecedented rate. Patients infected with the virus causing COVID-19, the severe acute respiratory syndrome coronavirus (SARS-CoV-2), vary in disease severity. An earlier study of 44,672 individuals with confirmed COVID-19 reported that 81% of the patients usually exhibit mild to moderate symptoms, while 14% show severe symptoms, and 5% experience critically serious disease [1]. Those with pre-existing medical conditions such as hypertension are particularly vulnerable to severe outcomes of COVID-19 [2]. Over 115 million people in the US have hypertension, and angiotensin converting enzyme inhibitors (ACEI) and angiotensin II receptor blockers (ARB) are frequently used for treatment [3]. Therefore, both ACEI and ARB are under scrutiny for their association with COVID-19 [4].

Both ACEI and ARB affect the renin-angiotensin-aldosterone system that involves the viral receptor protein, angiotensin-converting enzyme 2 (ACE2). The SARS-CoV-2 virus binds to this ACE2 receptor on target cells [5]. ACE2 mediates the conversion of angiotensin II to angiotensin 1–7, and angiotensin-converting enzyme (ACE) mediates conversion of angiotensin I to angiotensin II. ACEI directly inhibits ACE, and ARB blocks the interaction of angiotensin II with its receptor, angiotensin II type-1 receptor (AT1R). While ACEI and ARB exert their anti-hypertension effects through specific targets (ACE and AT1R), ACEI and ARB may have different effects on ACE2 and its interaction with the SARS-CoV-2 virus. Because hypertension is associated with COVID-19 outcomes [2] and because ACEI and ARB are widely used by patients with hypertension, it is extremely important to evaluate the benefits and risks associated with the use of ACEI or ARB during the COVID-19 pandemic.

Literature surveys have failed to identify any reports associating ACEI/ARB with a higher risk of COVID-19 infection, severity, or mortality [6, 7]. Meta-analysis of studies of ACEI/ARB usage among COVID-19 patients showed no significant increase in the risk of COVID-19 infection, but did show a decreased risk of severe COVID-19 and mortality in patients receiving ACEI/ARB therapy [8–10]. Furthermore, a meta-analysis of twenty-six studies involving 8,104 hypertensive ACEI/ARB users and 8,203 hypertensive non-ACEI/ARB users reported a significantly lower risk of mortality and a lower need for ventilator use among ACEI/ARB users [11]. We thus sought to investigate two classes of anti-hypertension medications, ACEI and ARB, for their different associations with COVID-19 occurrence and severity by using a large cohort of Veterans in the Department of Veterans Affairs (VA) COVID-19 Shared Data Resource.

## Methods

### Study population

This retrospective study of Veterans’ medical records was approved by the Bedford VA Healthcare System Institutional Review Board, and all data were fully anonymized before access. To access and analyze the VA data, we used the COVID-19 Shared Data Resource at the VA Informatics and Computing Infrastructure (VINCI) Resource Center, which is a partner of the Corporate Data Warehouse (CDW).

The COVID-19 Shared Data Resource contains information on all Veterans who received a COVID-19 nucleic acid real time polymerase chain reaction (RT-PCR) test (positive or negative) through the VA or who tested positive outside the VA, including information pertaining to the positive test recorded in the VA clinical notes using natural language processing.

We included in this study 377,206 Veterans who had a record of a COVID-19 test performed between February 28, 2020 and August 18, 2020. We identified 228,722 Veterans who had a history of hypertension in the two years prior to the index date, based on the International Classification of Diseases codes (Tenth Revision, Clinical Modification, ICD-10-CM). Participants with an invalid age for a Veteran (under 18 or over 105) were excluded.

### Definitions

Veterans positive for COVID-19 were defined by a positive nucleic acid RT-PCR test recorded in the database. The index date is defined as the date of the first positive or first negative test, or the date of the hospital admission that is closest (within 15 days) to the date of the first positive or negative test.

Medication exposure was defined by the prescription of two classes of anti-hypertension medications, ACEI and ARB, within two years of the index date. Veterans with prescriptions, identified using claims data indicating ACEI-only, ARB-only, or either ACEI or ARB, were compared to those without a prescription for either ACEI or ARB. Since concomitant use of ACEI and ARB is not recommended, the use of both of these drugs among Veterans with prescriptions for either ACEI or ARB may have been not concomitant use but a case of stopping the use of one and starting the use of the other.

Veterans were considered not exposed to ACEI or ARB if they had not been prescribed either ACEI or ARB within two years of the index date. Unexposed participants could have been prescribed other anti-hypertension medications, such as alpha blockers, beta blockers, or calcium channel blockers. Out of 87,085 Veterans with hypertension who had not been prescribed ACEI or ARB, 34.7% had been prescribed alpha blockers, 42.5% had been prescribed beta blockers, and 40.2% had been prescribed calcium channel blockers. In total, 73.3% had been prescribed at least one of these non-ACEI/ARB medications. The use of diuretics was not recorded in the COVID-19 Shared Data Resource. All medication use was evaluated based on prescriptions written in the 2-year period before the COVID-19 test date (index date).

The severity of COVID-19 was defined as mild, moderate, severe or critical stages of disease with a four-level severity measure. The most frequently used classification system for clinical prediction of COVID-19 severity is the World Health Organization’s criteria, which include mild, moderate, severe, and critical disease [12]. The claims data from the VA COVID-19 Shared Data Resource are consistent with and follow a system similar to the World Health Organization (WHO) criteria, with a similar classification scheme of COVID-19 severity. Among our COVID-19-positive Veterans who were alive 60 days after the index date, severity was classified using a four-level severity measure: level 1—mild disease and not hospitalized; level 2—moderate disease, hospitalized but not admitted to the intensive care unit (ICU) and not requiring mechanical ventilation; level 3—severe disease, admitted to ICU but without mechanical ventilation; level 4—critical disease requiring mechanical ventilation.

### Outcomes

We examined the associations of ACEI and ARB with COVID-19-positive test and severity outcomes based on observational clinical data from the VA. Two sets of comparisons were conducted: (1) users of ACEI only and users of ARB only versus those who had not used either ACEI or ARB in the two years prior to the index date, and (2) users of either ACEI or ARB versus those who had not used either ACEI or ARB.

First, we examined the association between ACEI/ARB use and a positive COVID-19 test result (as defined by an RT-PCR test). Second, we used hospitalization and mortality data from the 60 days following the index date as outcomes. Vital status was recorded in the COVID-19 Shared Data Resource. For analyses focusing on hospitalization and mortality, we excluded Veterans with missing hospitalization information and those who died as outpatients from our “non-hospitalized” group.

We examined the association of ACEI/ARB use with COVID-19 severity, using the previously defined four-level severity measure for COVID-19 infection. In these analyses, for the group of Veterans who were not hospitalized, we excluded those who died within 60 days after the index date and those who died after being hospitalized.

### Demographics and covariates

The demographic characteristics considered in this study included age at index date, race (Native American, Alaska Native, Asian, Black or African American, Native Hawaiian or other Pacific Islander, or White), sex, and ethnicity (Hispanic and non-Hispanic). We adjusted for comorbidities relevant to COVID-19, which were defined based on inpatient and outpatient ICD-10 codes from the two-year period preceding the index date and included diabetes, pulmonary disease, kidney disease, coronary atherosclerotic heart disease (CAHD), chronic liver disease, hyperlipidemia, human immunodeficiency virus (HIV), cancer, smoking status, chronic neurological disease, stroke, heart failure, asplenia, alcohol dependency, and drug dependency. We also adjusted for body mass index (BMI), which was measured at the index date and divided into quartiles, and the Charlson Comorbidity Index (CCI).

### Statistical analysis

We used logistic regression to estimate the odds ratio (OR) and the 95% confidence interval (CI) of the positive COVID-19 test among those prescribed ACEI only, ARB only, or either ACEI or ARB, compared to those not prescribed either ACEI or ARB. Because of the potential imbalance among covariates, we used propensity score weighting (PSW) to control for confounding in our sensitivity analyses. All analyses were adjusted for relevant covariates, including demographics (age, sex, race, ethnicity), comorbidities, BMI, and the CCI, as described above. To assess multi-collinearity among the dependent variables, we used the Generalized Variance Inflation Factor (GVIF) test with a threshold of 5 [13].

Among COVID-19-positive Veterans, we used nominal logistic regression with and without PSW to investigate the association of ACEI/ARB with hospitalization (vs. non-hospitalization, excluding outpatients who had died), mortality, and the four-level severity measure. In analyses focusing on severity, COVID-19-positive outpatients were used as the reference group (least severe).

We set the statistical significance (alpha) level at 0.05, and the maximum likelihood estimation was used to calculate the OR with 95% CI. To examine the robustness of the observed associations, we used a bootstrapping procedure to estimate the CI of the primary findings (regression coefficients) from logistic regression models. The bootstrapping procedure was used as a sensitivity analysis to control and check for the stability of the results, because it is asymptotically more accurate than the standard confidence intervals obtained from sample variance and assumptions of normality [14, 15].

## Results

### Characteristics of Veterans according to the use of ACEI and/or ARB

Our study population consisted of 65.5% White, 26.8% Black or African American, 0.8% Asian, 0.8% Native American or Alaska Native, 0.8% Native Hawaiian or other Pacific Islander, and 5.4% unreported race. Nearly 6.9% of the study population were Hispanic or Latino. The mean age (± SD) at the index date for Veterans with hypertension who had received the COVID-19 test was 66.8 ± 12.1 years. The mean age of the test-negative Veterans (66.9 ± 12.0 years) was fairly comparable to the test-positive Veterans (66.2 ± 13.2 years) (Table 1). The majority (93.0%) of the study cohort were male.

**Table 1:** Demographics of Veterans with hypertension tested for COVID-19 infection (negative or positive).

			Positive (N = 21420)	Negative (N = 207302)
Age at the index date, Mean (SD)			66.2 (13.2)	66.9 (12.0)
		Cohort N (%)	N (%)	N (%)
**Sex**	**Female**	**15,938 (7.0%)**	1,412 (6.6%)	14,526 (7.0%)
	**Male**	**212,784 (93.0%)**	20,008 (93.4%)	192,776 (93.0%)
**Race**	**Native American or Alaska Native**	**1,744 (0.8%)**	163 (0.8%)	1,581 (.8%)
	**Asian**	**1,801 (0.8%)**	125 (0.6%)	1,676 (0.8%)
	**Black or African American**	**61,220 (26.8%)**	8,474 (39.6%)	52,746 (25.4%)
	**Native Hawaiian or other Pacific Islander**	**1,832 (0.8%)**	180 (0.8%)	1,652 (0.8%)
	**Unknown**	**12,316 (5.4%)**	1,259 (5.9%)	11,057 (5.3%)
	**White**	**149,809 (65.5%)**	11,219 (52.4%)	138,590 (66.9%)
**Ethnicity**	**Hispanic or Latino**	**15,774 (6.9%)**	2,069 (9.7%)	13,705 (6.6%)
	**Not Hispanic or Latino**	**208,008 (90.9%)**	18,852 (88.0%)	189,156 (91.3%)
	**Unknown**	**4,940 (2.2%)**	499 (2.3%)	4,441 (2.1%)

Among all Veterans with hypertension, 50.8% of COVID-19-positive and 46.8% of COVID-19-negative Veterans had diabetes; 37.4% of COVID-19-positive and 46.3% of COVID-19-negative Veterans had pulmonary disease; 24.6% of COVID-19-positive and 28.1% of negative Veterans had acute kidney injury; 27.4% of COVID-19-positive and 33.0% of negative Veterans had coronary atherosclerosis or other heart diseases (Table 2).

**Table 2:** Comorbidities of Veterans with hypertension with or without COVID-19 infection.

			Positive(N = 21420)	Negative(N = 207302)
**Charlson Comorbidity Index (CCI), Mean (SD)**			2.9 (2.8)	3.4 (3.10)
**Quartile BMI, Mean (SD)**		**Q1 ≤ 25.3**	22.3 (2.5)	22.3 (2.4)
		**25.3 < Q2 ≤ 29.0**	27.3 (1.1)	27.2 (1.1)
		**29.0 < Q3 ≤ 33.4**	31.2 (1.3)	31.1 (1.2)
		**Q4 > 33.4**	38.6 (5.1)	38.6 (5.1)
		**Cohort N (%)**		
**Diabetes**	**Yes**	**107,813 (47.1%)**	10,883 (50.8%)	96,930 (46.8%)
	**No**	**120,909 (52.9%)**	10,537 (49.2%)	110,372 (53.2%)
**Pulmonary disease**	**Yes**	**104,086 (45.5%)**	8,014 (37.4%)	96,072 (46.3%)
	**No**	**124,636 (54.5%)**	13,406 (62.6%)	111,230 (53.7%)
**Kidney disease**	**Acute kidney injury**	**63,455 (27.7%)**	5,276 (24.6%)	58,179 (28.1%)
	**Severe kidney disease**	**5,745 (2.5%)**	548 (2.6%)	5,197 (2.5%)
	**Normal & chronic stable**	**130,762 (57.2%)**	12,410 (57.9%)	118,352 (57.1%)
	**Unknown**	**28,760 (12.6%)**	3,186 (14.9%)	25,574 (12.3%)
**Coronary atherosclerosis and other heart disease**	**Yes**	**74,169 (32.4%)**	5,867 (27.4%)	68,302 (33.0%)
	**No**	**154,553 (67.6%)**	15,553 (72.6%)	139,000 (67.1%)
**Chronic liver disease**	**Yes**	**10,921 (4.8%)**	704 (3.3%)	10,217 (4.9%)
	**No**	**217,801 (95.2%)**	20,716 (96.7%)	197,085 (95.1%)
**Hyperlipidemia**	**Yes**	**165,895 (72.5%)**	15,232 (71.1%)	150,663 (72.7%)
	**No**	**62,827 (27.5%)**	6,188 (28.9%)	56,639 (27.3%)
**Human immunodeficiency virus**	**Yes**	**2,500 (1.1%)**	273 (1.3%)	2,227 (1.1%)
	**No**	**226,222 (98.9%)**	21,147 (98.7%)	205,075 (98.9%)
**Cancer**	**Yes**	**73,714 (32.2%)**	5,284 (24.7%)	68,430 (33.0%)
	**No**	**155,008 (67.8%)**	16,136 (75.3%)	138,872 (67.0%)
**Smoke**	**Current smoker**	**46,219 (20.2%)**	2,330 (10.9%)	43,889 (21.2%)
	**Former smoker**	**103,097 (45.1%)**	9,768 (45.6%)	93,329 (45.0%)
	**Never smoker**	**71,455 (31.2%)**	8,413 (39.3%)	63,042 (30.4%)
	**Unknown**	**7,951 (3.5%)**	909 (4.2%)	7,042 (3.4%)
**Chronic neurological Disease**	**Yes**	**14,957 (6.5%)**	1,661 (7.8%)	13,296 (6.4%)
	**No**	**213,765 (93.5%)**	19,759 (92.3%)	194,006 (93.6%)
**Stroke**	**Yes**	**24,073 (10.5%)**	2,258 (10.5%)	21,815 (10.5%)
	**No**	**204,649 (89.5%)**	19,162 (89.5%)	185,487 (89.5%)
**Asplenia**	**Yes**	**1,038 (0.5%)**	96 (.5%)	942 (.5%)
	**No**	**227,684 (99.5%)**	21,324 (99.5%)	206,360 (99.5%)
**Alcohol dependency**	**Yes**	**33,767 (14.8%)**	2,313 (10.8%)	31,454 (15.2%)
	**No**	**194,955 (85.2%)**	19,107 (89.2%)	175,848 (84.8%)
**Drug dependency**	**Yes**	**18,554 (8.1%)**	1,150 (5.4%)	17,404 (8.4%)
	**No**	**210,168 (91.9%)**	20,270 (94.6%)	189,898 (91.6%)
**Heart failure**	**Yes**	**42,565 (18.6%)**	3,326 (15.5%)	39,239 (18.9%)
	**No**	**186,157 (81.4%)**	18,094 (84.5%)	168,063 (81.1%)

Among 228,722 COVID-19-tested Veterans with hypertension, we identified 94,101 Veterans who used ACEI only; 39,281 who used ARB only; 141,637 who used ACEI or ARB; and 87,085 Veterans not using either ACEI or ARB. Unexposed participants could have been prescribed other anti-hypertension medications such as alpha blockers, beta blockers, or calcium channel blockers (S1 Table). The mean age at the index date for ARB-only users (68.0 ± 10.1 years) was comparable to that of ACEI-only users (66.8 ± 11.4 years) and non-ACEI/ARB users (66.3 ± 13.3 years) (Table 3).

**Table 3:** Characteristics of ACEI/ARB use in Veterans with hypertension.

		No ACEI, no ARB(N = 87085)	ACEI only (N = 94101)	ARB only (N = 39281)	ACEI or ARB (N = 141637)
Age at index date, Mean (SD)		66.3 (13.3)	66.8 (11.4)	68.0 (10.1)	67.2 (11.3)
		No ACEI No ARB	ACEI-only	ARB-only	ACEI or ARB
**Sex**	**Female**	7,681 (8.8%)	4,769 (5.1%)	2,953 (7.5%)	8,257 (5.8%)
	**Male**	79,404 (91.2%)	89,332 (94.9%)	36,328 (92.5%)	133,380 (94.2%)
**Race**	**Native American or Alaska Native**	665 (0.8%)	773 (0.8%)	257 (0.7%)	1,079 (0.8%)
	**Asian**	676 (0.8%)	584 (0.6%)	469 (1.2%)	1,125 (0.8%)
	**Black or African American**	24,646 (28.3%)	23,095 (24.5%)	11,115 (28.3%)	36,574 (25.8%)
	**Native Hawaiian or Other Pacific Islander**	649 (0.8%)	814 (0.9%)	309 (0.8%)	1,183 (0.8%)
	**Unknown**	4,805 (5.5%)	5,048 (5.4%)	2,051 (5.2%)	7,511 (5.3%)
	**White**	55,644 (63.9%)	63,787 (67.8%)	25,080 (63.9%)	94,165 (66.5%)
**Ethnicity**	**Hispanic or Latino**	5,439 (6.3%)	6,818 (7.3%)	2,846 (7.3%)	10,335 (7.3)
	**Not Hispanic or Latino**	79,692 (91.5%)	85,252 (90.6%)	35,627 (90.7%)	128,316 (90.6%)
	**Unknown**	1,954 (2.2%)	2,031 (2.2%)	808 (2.1%)	2,986 (2.1%)

Out of our Veteran population, 5.1% ACEI-only users were female compared to 7.5% for ARB-only users, and 94.9% ACEI-only users were male compared to 92.5% for ARB-only users (Table 3). Overall, all racial groups in our study had comparable proportions of ACEI-only users and ARB-only users (Table 3).

### Association of ACEI use with reduced COVID-19-positive test outcomes

**Fig 1:**
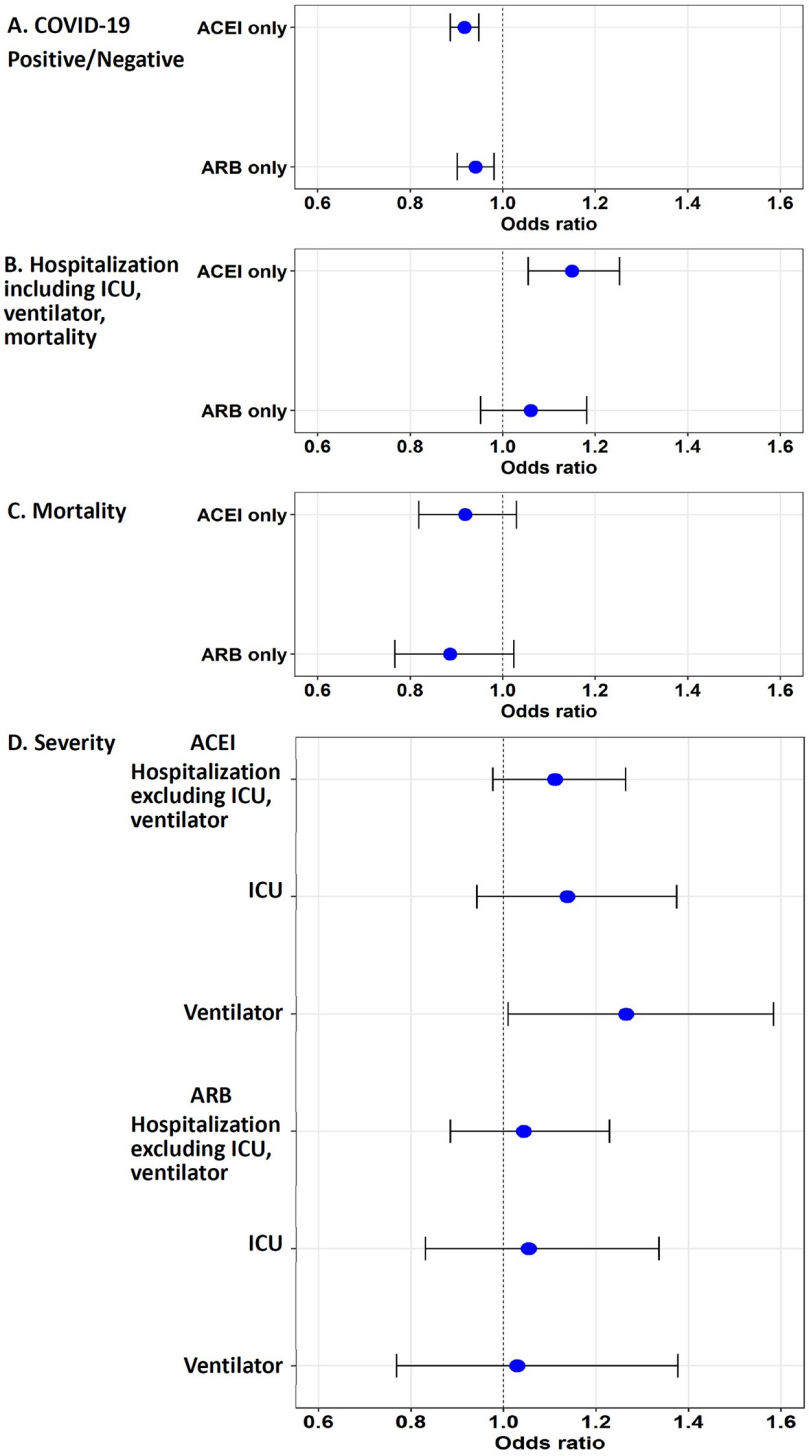
Odds ratios of COVID-19 infection and severity in Veterans with records of using ACEI only or ARB only. Veterans with records of having been prescribed ACEI only or ARB only were compared to those with no record of having been prescribed ACEI or ARB using a logistic regression model. **A**. The use of ACEI only is associated with reduced odds of positive COVID-19 test. **B**. Among COVID-19-positive Veterans with hypertension, the use of ACEI is associated with increased odds of hospitalization (including all inpatients admitted to ICU, using mechanical ventilation, or having died). **C**. Use of ACEI/ARB is not significantly associated with mortality. **D**. No significant association was observed between ACEI/ARB use and hospitalization (excluding those admitted to ICU or using ventilators) or ICU admission; record of ACEI prescription is associated with an increase in the use of mechanical ventilation.

Veterans who used ACEI only in the 2 years before the index date had 8.2% lower adjusted odds of a positive COVID-19 test (OR = 0.917, (0.887, 0.948), *p* < 0.001) compared to Veterans who did not use ACEI or ARB (Table 4, Fig 1A). In analyses applying PSW, the adjusted OR for ACEI users was comparable (PSW OR = 0.926, (0.894, 0.958), *p* < 0.001) (S2 Table).

**Table 4:** Association of ACEI/ARB alone with COVID-19 infection and severity.

Outcome variable		ACEI/ARB	Odds	95% CI	p-value
**COVID-19 infection (Positive/Negative)**		ACEI only	0.917	(0.887, 0.948)	<0.001
		ARB only	0.941	(0.902, 0.982)	0.005
**Hospitalization (including ICU, ventilator, or death)**		ACEI only	1.150	(1.055, 1.253)	0.001
		ARB only	1.061	(0.952, 1.182)	0.288
**Death**		ACEI only	0.919	(0.819, 1.030)	0.147
		ARB only	0.886	(0.767, 1.024)	0.102
**Severity**	**Hospitalization (excluding ICU or ventilator)**	ACEI only	1.111	(0.977, 1.264)	0.107
	**ICU**	ACEI only	1.138	(0.942, 1.374)	0.180
	**Ventilator**	ACEI only	1.265	(1.010, 1.584)	0.041
	**Hospitalization (excluding ICU or ventilator)**	ARB only	1.043	(0.885, 1.229)	0.614
	**ICU**	ARB only	1.054	(0.831, 1.336)	0.666
	**Ventilator**	ARB only	1.029	(0.769, 1.377)	0.845

Note: Results were adjusted by race, sex, ethnicity, diabetes, pulmonary disease, kidney disease, coronary atherosclerotic heart disease (CAHD), chronic liver disease, hyperlipidemia, HIV, cancer, smoking status, chronic neurological disease, stroke, heart failure, asplenia, alcohol dependency, drug dependency, Charlson Comorbidity Index (CCI), and body mass index.

ARB use was associated with reduced odds of a positive COVID-19 test outcome in multivariate logistic regression analyses (OR = 0.941, (0.902, 0.981), *p* = 0.005) (Table 4). This association was attenuated after PSW (PSW OR = 0.958, (0.916, 1.001), *p* = 0.058) (S2 Table).

**Fig 2:**
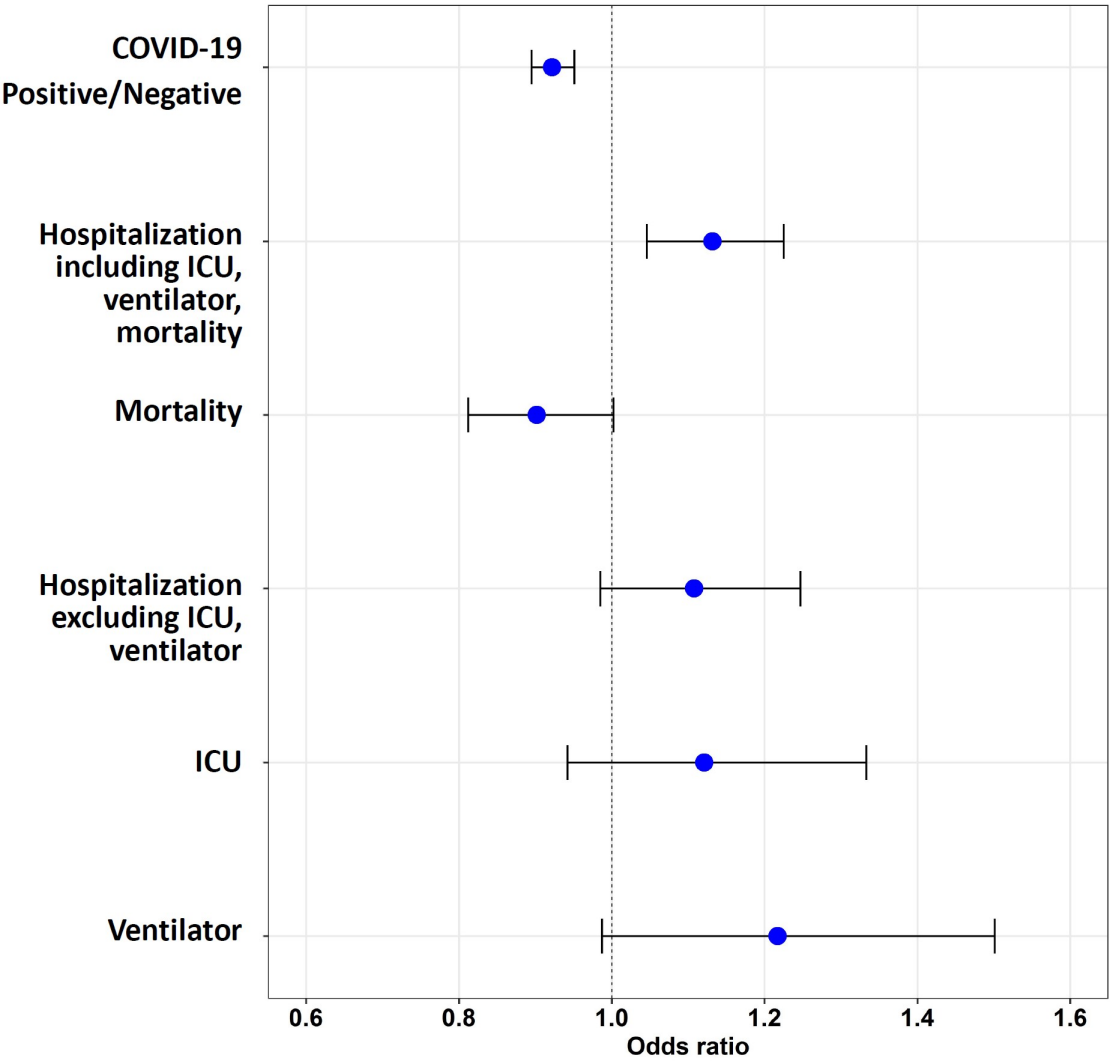
Odds ratios of COVID-19 infection and severity in Veterans with records of using ACEI or ARB. Veterans with records of having been prescribed either ACEI or ARB were compared to those with no record of having been prescribed ACEI or ARB using a logistic regression model. Use of ACEI or ARB is associated with reduced odds of a positive COVID-19 test. Among COVID-19-positive Veterans with hypertension, the use of ACEI or ARB is associated with increased odds of hospitalization (including all inpatients admitted to ICU, using mechanical ventilation, or having died). The use of ACEI or ARB was not associated with mortality. No significant association was observed between ACEI/ARB use and hospitalization (excluding those admitted to ICU or using ventilators), ICU admission, or the use of mechanical ventilation.

The use of ACEI or ARB, compared to the use of neither medication, was also associated with a reduced odds of a COVID-19 positive test in logistic regression both without PSW (OR = 0.922, (0.895, 0.951), *p* < 0.001) (Table 5, Fig 2) and with PSW models (PSW OR = 0.934, (0.905, 0.964), *p* < 0.001) (S3 Table). Generalized Variance Inflation Factor (GVIF) was less than 5, indicating no multi-collinearity concerns (S4 Table) [13].

**Table 5:** Association of ACEI or ARB use with COVID-19 infection and severity.

Outcome variable			Odds	95% CI	P-value
**COVID-19 infection (Positive/Negative)**		ACEI or ARB	0.922	(0.895, 0.951)	<0.001
**Hospitalization (including ICU, ventilator, or death)**		ACEI or ARB	1.132	(1.046, 1.225)	0.002
**Death**		ACEI or ARB	0.902	(0.812, 1.002)	0.054
**Severity**	**Hospitalization (excluding ICU or ventilator)**	ACEI or ARB	1.108	(0.985, 1.247)	0.088
	**ICU**	ACEI or ARB	1.121	(0.942, 1.333)	0.198
	**Ventilator**	ACEI or ARB	1.217	(0.987, 1.501)	0.067

Note: Results were adjusted by race, sex, ethnicity, diabetes, pulmonary disease, kidney disease, coronary atherosclerotic heart disease (CAHD), chronic liver disease, hyperlipidemia, HIV, cancer, smoking status, chronic neurological disease, stroke, heart failure, asplenia, alcohol dependency, drug dependency, Charlson Comorbidity Index (CCI), and body mass index.

### The use of ACEI, but not ARB, is associated with increased odds of hospitalization in COVID-19-positive Veterans with hypertension

After 7,956 Veterans with missing hospitalization information were excluded from the analysis, out of 13,464 COVID-19-positive Veterans, 6,404 had not been admitted to the hospital (excluding 888 outpatients who had died within 60 days after the index date), and 6,172 Veterans had been admitted to the hospital. These hospitalized Veterans included those admitted to the ICU (680 Veterans), those requiring mechanical ventilators (462 Veterans), those who died after hospitalization (1,044 Veterans), and those with missing ICU/ventilation information (2,303 Veterans). Among the COVID-19-positive Veterans, the use of ACEI during the past two years was associated with a significant increase in the odds of hospitalization in both logistic regression (OR = 1.150, (1.055, 1.253), *p* = 0.001) and PSW models (PSW OR = 1.124, (1.028, 1.228), *p* = 0.010), compared to the Veterans who had no record of using either ACEI or ARB (Fig 1B, Table 4, S2 Table). No significant increase in the odds of hospitalization was observed for those using ARB (Fig 1B, Table 4). When comparing the Veterans using either ACEI or ARB to non-users, the odds of being hospitalized were significantly elevated (OR = 1.132, (1.046, 1.225), *p* = 0.002; PSW OR = 1.112, (1.025, 1.206), *p* = 0.011) (Fig 2, Table 5, S3 Table).

### The use of ACEI or ARB and mortality among COVID-19-positive Veterans with hypertension

According to death records, 2,006 of the 21,420 COVID-19-positive Veterans died within 60 days after the index date. We did not observe a statistically significant association between ACEI-only use and mortality compared to the Veterans who had no record of using either ACEI or ARB in nominal logistic regression (OR = 0.919, (0.819, 1.030), *p* = 0.147) or PSW model (OR = 0.905, (0.803, 1.020), *p* = 0.102); nor was there a statistically significant association between ARB-only use and mortality in logistic regression (OR = 0.886, (0.767, 1.024), *p* = 0.102) or PSW model (OR = 0.896, (0.769, 1.045), *p* = 0.161) (Fig 1C, Table 4, S2 Table). Similarly, we did not observe a significant association between use of ACEI or ARB and the odds of death in logistic regression (OR = 0.902, (0.812, 1.002), *p* = 0.054) or PSW model (OR = 0.893, (0.801, 0.997), *p* = 0.043) (Fig 2, Table 5, S3 Table).

### Association of ACEI, but not ARB, with increased odds of using mechanical ventilation

To examine the association of ACEI and ARB use with the four-level severity measure previously defined, we compared the cohort of 6,404 Veterans who had not been admitted to the hospital within 60 days after the index date to Veterans who had been hospitalized (1,683 Veterans), had been admitted to the ICU (680 Veterans), or required mechanical ventilation (462 Veterans). The use of ACEI or ARB was not associated with hospitalization or admission to the ICU (Fig 1D, Table 4). Interestingly, we found that the use of ACEI, but not ARB, was associated with increased odds of using mechanical ventilation (OR = 1.265, (1.010, 1.584), *p* = 0.041); PSW model (OR = 1.210, (1.053, 1.390), *p* = 0.007) (Fig 1D, Table 4, S2 Table). We did not observe an association between ARB use and mechanical ventilation (Fig 1D, Table 4). When either ACEI or ARB use was compared to non-use, no relationship to severity was observed (Fig 2, Table 5).

Sensitivity analyses bootstrapping the confidence intervals for logistic regression were comparable with our primary findings (S5 Table), supporting the robustness of the associations reported in our analyses.

## Discussion

The mechanisms of action of ACEI and ARB on targets related to the SARS-CoV-2 virus receptor protein ACE2 potentially implicate these medications in COVID-19 risk and outcomes. The VA COVID-19 Shared Data Resource is a unique platform providing a comprehensive dataset for multi-faceted analyses of COVID-19-related clinical medical records. This study is one of the first attempts to distinguish the long-term use of ACEI from that of ARB and from their combined use, taking advantage of extensive data on COVID-19-related comorbidities from a large cohort of 228,722 Veterans with hypertension. Our approach has several strengths and is unique compared to previously published studies.

This study focuses on a large population of Veterans who have had ready access to healthcare, with medical records collected over a long duration available in one of the nation’s largest integrated healthcare systems. This sample consists of Veterans with high prevalence of comorbid conditions compared to the general population. These Veterans represent a variety of races, ethnicities, and sociodemographic backgrounds. For example, a quarter of Veteran with hypertension are African American. The presence of over 20,000 COVID-19-positive Veterans in our cohort makes it one of the largest collections of COVID-19-relevant medical records. Thus, outcomes from our analyses are likely representative across various races and ethnicities. Previous studies on a larger cohort of 43,000 Italian patients revealed a modest statistically significant increase in mortality risk for any anti-hypertension drug use compared to non-use. In that study, ACEI/ARB use was not associated with the risk of all-cause mortality, compared with the use of calcium channel blockers [16]. Studies based on the Danish COVID-19 registries of 4,480 subjects, with data collected from February 22 to May 4, 2020, suggested that prior use of ACEI/ARB was not associated with COVID-19 infection in the nested case-control susceptibility analysis. This retrospective cohort study also failed to detect any association of ACEI/ARB with death or with a composite outcome of death/severity of COVID-19 [17]. Similar studies across the globe with smaller sample sizes support little or no association between ACEI/ARB and the severity of disease or mortality among those patients with hypertension or other comorbidities. Studies of 1,178 COVID-19-positive hospitalized patients in China [18], 1,200 inpatients in the United Kingdom [19], 5,179 patients in Korea [20], 111 patients in France [21], 1,603 patients in Italy [22], 338 patients in Saudi Arabia [23], 659 patients in Brazil [24], and 1,449 patients in the US [25] revealed no significant associations.

Our study design allowed us to differentiate ACEI use from ARB use by utilizing claims data from Veterans with prescriptions for ACEI only, for ARB only, or for either ACEI or ARB, compared to Veterans with prescriptions for neither ACEI nor ARB. This approach allowed us to address specific differentiating questions regarding the association between ACEI/ARB and COVID-19 infection/severity. We found that Veterans taking ACEI only had reduced COVID-19 occurrence. On the other hand, the association of ARB use with a positive COVID-19 test was not significant after propensity score weighting. Thus, our study suggests that the use of ACEI may benefit Veterans with hypertension.

We used a four-level COVID-19 severity measure and did not observe a consistent association between the use of either ACEI or ARB and disease severity. A previous study of 590 COVID-19 patients (78 ACEI/ARB users vs. 512 non-users) from a single center did not reveal any significant association between ACEI/ARB use and hospitalization, ICU admissions, mechanical ventilation, length of hospital stay, use of inotropes, or all-cause mortality [26]. A multi-center study of 338 patients yielded similar results: no significant associations between ACEI/ARB use and hospitalization, ICU admission, mechanical ventilation, or mortality. However, the same study found that maintaining ACEI/ARB use during hospitalization significantly lowered the likelihood of death [23]. A study of 614 hospitalized COVID-19 patients with hypertension did not reveal a significant association between ACEI/ARB use and ICU/mortality. However, inpatients who continued ACEI/ARB use had a significant reduction in ICU admission and mortality [27]. There was no significant association between the dose of ACEI/ARB and COVID-19 infection or hospitalization in a cohort of 826 COVID-19-positive patients [28].

In our study, we observed a statistically significant association of ACEI use and mechanical ventilation. The finding that exposure to ACEI is associated with increased odds of using mechanical ventilators among COVID-19-positive inpatients with hypertension may be due to a complex relationship of proximal factors, including unmeasured clinical variables that may confound these results. Since ACEI use was associated with increased odds of using mechanical ventilators but not of ICU admission, we do not have sufficient evidence from other clinical variables to support a consistent association between ACEI/ARB use and COVID-19 severity.

ACEI and ARB exhibited different associations with a positive COVID-19 test and severity outcomes, possibly reflecting their different biological targets at the molecular level. ACEI inhibits ACE, and ARB blocks angiotensin II receptor AT1R; both pathways are balanced by ACE2, the SARS-CoV-2 virus receptor. SARS-CoV-2 virus infection triggers a variety of physiological responses. While earlier studies showed that treatment with ACEI or ARB leads to up-regulation of ACE2 in patients with type 1 or 2 diabetes [5] or in patients with hypertension [29], a recent study suggests that ACEI/ARB do not increase the expression of the ciliary ACE2 receptor, and thus, they may not enhance susceptibility to SARS-CoV-2 infection [30]. This is supported by human studies lacking clear evidence linking ACEI and ARB to increased severity of COVID-19 [31, 32]. In COVID-19 patients with hypertension, it is not clear whether ACEI use changes the levels of ACE2 and gives SARS-CoV-2 more cellular entry points, which causes more damage. In mice, SARS-CoV (not SARS-CoV-2) reduces ACE2 abundance and causes acute lung failure [33]. Treating human cellular organoids with recombinant ACE2 protein reduces the viral load of SARS-CoV-2 [14], and a similar effect was reported with ARB [34]. Therefore, the levels of ACE2 may vary in the presence of ACEI or ARB, leading to different physiological consequences.

Our study has a number of limitations. Like other observational studies, our investigation is subject to bias and confounding. Due to the retrospective, observational nature of our study and to the potential for selection and other biases, our ability to draw causal inferences is limited. The majority of our subjects (93.0%) were male, due to the overall nature of the population of Veterans who had served in the US military. While we have adjusted for sex in our statistical analyses, our study lacked sufficient power to conduct sex-stratified analyses. Second, our study participants took COVID-19 tests voluntarily and the various social and behavioral reasons associated with testing were not measured in our dataset and could not be adjusted for in our analyses. The decision to test for COVID-19 may be driven by factors such as symptomology, risk perceptions, health conditions, behavioral attitudes, and employment requirements, among others. Third, our analyses focused on prescribed medications as a measure of exposure and we made the assumption that Veterans prescribed the drugs of interest (ACEI or ARB) were actually taking the medication. Using only prescriptions reported in claims data, it is not possible for us to confirm patient compliance to ACEI or ARB therapy. The effects of a lack of adherence to the ACEI and ARB medication prescriptions are likely to bias our results towards the null hypothesis, so the results reported in this paper are likely to be conservative. Fourth, the choice of ACEI or ARB by the provider may be based on an individual’s long hypertension history, and we have only retrieved data for the most recent pre-existing conditions from our COVID-19 Shared Data Resource. Fifth, our observation of the significant association between the use of ACEI and mechanical ventilators should be interpreted with caution, as we did not find a similar association between ACEI use and ICU admission.

While most studies have reported no association between the use of ACEI or ARB and the occurrence of COVID-19 [16–25], we found that the presence of a claim record of an ACEI prescription was associated with an 8% reduction in the OR of a COVID-19 infection. While this finding warrants further investigation, we do not believe it justifies a change in clinical practice. The lack of association between ACEI or ARB and COVID-19 severity both in our study and in earlier publications [23, 26–28] further supports the notion that prescription of ACEI or ARB should not be discontinued in light of the rapidly changing pandemic.

In summary, based on our findings, ACEI use was associated with decreased odds of testing positive for COVID-19. The risks versus benefits of using one class of anti-hypertension medications over another should always be based on an individual’s medical history and preferences, and the results from our observational study need to be further validated in other larger cohorts outside the VA.

## Supporting information

S1 Table Characterization of Veterans with hypertension prescribed alpha blockers, beta blockers, or calcium channel blockers, but not ACEI or ARB. (DOCX)

S2 Table Association of ACEI/ARB use with COVID-19 infection and severity (PS logistic). (DOCX)

S3 Table Association of use of ACEI or ARB with COVID-19 infection and severity (PS logistic). (DOCX)

S4 Table Generalized Variance Inflation Factor (GVIF) test for multicollinearity diagnostics. (DOCX)

S5 Table Bootstrapping CI for logistic regression odds ratio. (DOCX)
