# Supplementary material for: The association of COVID-19 occurrence and severity with the use of angiotensin converting enzyme inhibitors or angiotensin-II receptor blockers in patients with hypertension
Source: PLoS One. 2021 Mar 18;16(3):e0248652. doi: 10.1371/journal.pone.0248652 (PMC7971559; doi:10.1371/journal.pone.0248652)
Supplement: S1 Table — (DOCX) [file pone.0248652.s001.docx]

**S1 Table. Characterization of Veterans with hypertension prescribed alpha blockers, beta blockers, or calcium channel blockers, but not ACEI or ARB.**

|  | **No ACEI, no ARB (N=87085)** | |
| --- | --- | --- |
| **Antihypertension medications** | **N** | **%** |
| **Alpha blocker** | **30,226** | **34.7%** |
| **Beta blocker** | **37,018** | **42.5%** |
| **Calcium channel blocker** | **34,975** | **40.2%** |
| **Any of the above medications** | **63,832** | **73.3%** |
