# Supplementary material for: The association of COVID-19 occurrence and severity with the use of angiotensin converting enzyme inhibitors or angiotensin-II receptor blockers in patients with hypertension
Source: PLoS One. 2021 Mar 18;16(3):e0248652. doi: 10.1371/journal.pone.0248652 (PMC7971559; doi:10.1371/journal.pone.0248652)
Supplement: S3 Table — (DOCX) [file pone.0248652.s003.docx]

**S3 Table. Association of the use of ACEI or ARB with COVID-19 infection and severity (PS logistic).**

| **Outcome variables** | | **Odds** | **95% CI** | **p-value** |
| --- | --- | --- | --- | --- |
| **COVID-19 infection (Positive/Negative)** | | 0.934 | (0.905, 0.964) | <0.001 |
| **Hospitalization (including ICU, ventilator, or death)** | | 1.112 | (1.025, 1.206) | 0.011 |
| **Death** | | 0.893 | (0.801, 0.997) | 0.043 |
| **Severity** | **Hospitalization (excluding ICU or ventilator)** | 1.054 | (0.974, 1.141) | 0.195 |
|  | **ICU** | 1.082 | (0.963, 1.215) | 0.186 |
|  | **Ventilator** | 1.138 | (0.992, 1.307) | 0.066 |

Note: Results were adjusted by race, sex, ethnicity, diabetes, pulmonary disease, kidney disease, coronary atherosclerotic heart disease (CAHD), chronic liver disease, hyperlipidemia, HIV, cancer, smoking status, chronic neurological disease, stroke, heart failure, asplenia, alcohol dependency, drug dependency, Charlson Comorbidity Index (CCI), and body mass index.
