# Supplementary material for: The association of COVID-19 occurrence and severity with the use of angiotensin converting enzyme inhibitors or angiotensin-II receptor blockers in patients with hypertension
Source: PLoS One. 2021 Mar 18;16(3):e0248652. doi: 10.1371/journal.pone.0248652 (PMC7971559; doi:10.1371/journal.pone.0248652)
Supplement: S4 Table — (DOCX) [file pone.0248652.s004.docx]

**S4 Table. Generalized Variance Inflation Factor (GVIF) test for multicollinearity diagnostics.**

|  | **GVIF** | **Standardized GVIF** |
| --- | --- | --- |
| Medication (ACEI, ARB) | 1.110 | 1.026 |
| Sex | 1.072 | 1.035 |
| Race | 1.325 | 1.029 |
| Ethnicity | 1.225 | 1.052 |
| Age at index date | 1.440 | 1.200 |
| CCI | 2.705 | 1.645 |
| Diabetes | 1.536 | 1.239 |
| Quartile BMI | 1.223 | 1.106 |
| Pulmonary disease | 1.136 | 1.066 |
| Kidney disease | 1.345 | 1.051 |
| Heart failure | 1.378 | 1.174 |
| CAHD | 1.268 | 1.126 |
| Chronic liver disease | 1.053 | 1.026 |
| Hyperlipidemia | 1.125 | 1.061 |
| Human immunodeficiency virus | 1.145 | 1.070 |
| Cancer | 1.213 | 1.102 |
| Smoke | 1.140 | 1.022 |
| Chronic neurological disease | 1.037 | 1.018 |
| Stroke | 1.118 | 1.057 |
| Asplenia | 1.004 | 1.002 |
| Alcohol dependency | 1.197 | 1.094 |
| Drug dependency | 1.160 | 1.077 |

Note: Standardized GVIF represents GVIF^(1/(2*Df)), where the Df stands for “degree of freedom.”
