# Supplementary material for: The association of COVID-19 occurrence and severity with the use of angiotensin converting enzyme inhibitors or angiotensin-II receptor blockers in patients with hypertension
Source: PLoS One. 2021 Mar 18;16(3):e0248652. doi: 10.1371/journal.pone.0248652 (PMC7971559; doi:10.1371/journal.pone.0248652)
Supplement: S5 Table — (DOCX) [file pone.0248652.s005.docx]

**S5 Table. Bootstrapping CI for logistic regression odds ratio**

| **Outcome variables** | | **ACEI/ARB** | **2.5%** | **97.5%** | **Median** |
| --- | --- | --- | --- | --- | --- |
| **COVID-19 infection (Positive/Negative)** | | ACEI only | 0.917 | 0.885 | 0.946 |
|  |  | ARB only | 0.942 | 0.901 | 0.984 |
| **Hospitalization (including ICU, ventilator, or death)** | | ACEI only | 1.150 | 1.053 | 1.248 |
|  |  | ARB only | 1.058 | 0.946 | 1.185 |
| **Severity** | **Hospitalization (excluding ICU or ventilator)** | ACEI only | 1.004 | 1.107 | 1.223 |
|  | **ICU** | ACEI only | 0.982 | 1.131 | 1.311 |
|  | **Ventilator** | ACEI only | 1.062 | 1.255 | 1.507 |

Note: Results were adjusted by race, sex, ethnicity, diabetes, pulmonary disease, kidney disease, coronary atherosclerotic heart disease (CAHD), chronic liver disease, hyperlipidemia, HIV, cancer, smoking status, chronic neurological disease, stroke, heart failure, asplenia, alcohol dependency, drug dependency, Charlson Comorbidity Index (CCI), and body mass index.
